# A propensity score-matched analysis of the impact of statin therapy on the outcomes of patients with non-small-cell lung cancer receiving anti-PD-1 monotherapy: a multicenter retrospective study

**DOI:** 10.1186/s12885-022-09385-8

**Published:** 2022-05-06

**Authors:** Kazuki Takada, Mototsugu Shimokawa, Shinkichi Takamori, Shinichiro Shimamatsu, Fumihiko Hirai, Tetsuzo Tagawa, Tatsuro Okamoto, Motoharu Hamatake, Yuko Tsuchiya-Kawano, Kohei Otsubo, Koji Inoue, Yasuto Yoneshima, Kentaro Tanaka, Isamu Okamoto, Yoichi Nakanishi, Masaki Mori

**Affiliations:** 1 Department of Thoracic Surgery, Kitakyushu Municipal Medical Center, 2-1-1 Bashaku, Kokurakita-ku, Kitakyushu, Fukuoka, 802-8561 Japan; 2 Department of Biostatistics, Yamaguchi University Graduate School of Medicine, 1-1-1 Minamikogushi, Ube, Yamaguchi, 755-8505 Japan; 3 Clinical Research Institute, National Hospital Organization Kyushu Cancer Center, 3-1-1 Notame, Minami-ku, Fukuoka, 811-1395 Japan; 4 Department of Thoracic Oncology, National Hospital Organization Kyushu Cancer Center, 3-1-1 Notame, Minami-ku, Fukuoka, 811-1395 Japan; 5 Department of Surgery and Science, Graduate School of Medical Sciences, Kyushu University, 3-1-1 Maidashi, Higashi-ku, Fukuoka, 812-8582 Japan; 6 Department of Respiratory Medicine, Kitakyushu Municipal Medical Center, 2-1-1 Bashaku, Kokurakita-ku, Kitakyushu, Fukuoka, 802-8561 Japan; 7 Research Institute for Diseases of the Chest, Graduate School of Medical Sciences, Kyushu University, 3-1-1 Maidashi, Higashi-ku, Fukuoka, 812-8582 Japan

**Keywords:** Nivolumab, Non-small-cell lung cancer, Pembrolizumab, Statin, Prognostic factor

## Abstract

**Background:**

Many studies have recently reported the association of concomitant medications with the response and survival in patients with non-small-cell lung cancer (NSCLC) treated with cancer immunotherapy. However, the clinical impact of statin therapy on the outcome of cancer immunotherapy in patients with NSCLC is poorly understood.

**Methods:**

In our database, we retrospectively identified and enrolled 390 patients with advanced or recurrent NSCLC who were treated with anti-programmed cell death-1 (PD-1) monotherapy in clinical practice between January 2016 and December 2019 at 3 medical centers in Japan to examine the clinical impact of statin therapy on the survival of patients with NSCLC receiving anti-PD-1 monotherapy. A propensity score-matched analysis was conducted to minimize the bias arising from the patients’ backgrounds.

**Results:**

The Kaplan–Meier curves of the propensity score-matched cohort showed that the overall survival (OS), but not the progression-free survival (PFS), was significantly longer in patients receiving statin therapy. However, a Cox regression analysis in the propensity score-matched cohort revealed that statin therapy was not an independent favorable prognostic factor, although it tended to be correlated with a favorable outcome.

**Conclusions:**

Statin therapy may be a combination tool for cancer immunotherapy in patients with NSCLC. These findings should be validated in further prospective studies with larger sample sizes.

**Supplementary Information:**

The online version contains supplementary material available at 10.1186/s12885-022-09385-8.

## Background

Immune checkpoint inhibitors (ICIs) targeting the programmed cell death-1 (PD-1)/programmed cell death-ligand 1 (PD-L1) pathway are the standard therapeutic options for cancer patients. However, many previous reports have revealed that a minority of patients with non-small-cell lung cancer (NSCLC) responds to ICIs in the clinical setting [1–3]. Therefore, we need to identify strategies to improve the efficacy of cancer immunotherapy. Recently, a number of studies have described concomitant medications associated with the response and survival in patients with NSCLC treated with cancer immunotherapy, including antibiotics, proton pump inhibitors, probiotics, beta blockers, and metformin [4–9], so there may be other drugs that improve the outcome of patients with NSCLC receiving cancer immunotherapy.

Statins are widely prescribed cholesterol-lowering drugs that inhibit the conversion of 3-hydroxy-3-methylglutaryl coenzyme A (HMG-CoA) to mevalonate by inhibiting the rate-limiting enzyme of the mevalonate pathway, which supports tumorigenesis and is deregulated in cancers [10]. Many retrospective studies have shown that statin use is associated with a reduced cancer risk and recurrence or cancer-specific mortality [11–16]. Furthermore, statins are also expected to improve the effect of cancer immunotherapy according to a previous report [17]. In this report, high cholesterol in tumor-infiltrating CD8+ T cells was associated with high expression of immune checkpoint factors and caused T cell exhaustion, while reducing the cholesterol levels restored the T cell function of anti-cancer activity. These findings could lead to the potential for statin therapy to be applied as a combination tool for cancer immunotherapy. Cantini et al. recently revealed that statin use was significantly associated with a better tumor response and longer progression-free survival (PFS) in patients with NSCLC treated with PD-1 inhibitors in an intensity-dependent manner [18]. Moreover, Omori et al. also indicated that statin use was significantly associated with the improved response rates and the prolonged time-to-treatment failure in NSCLC patients treated with nivolumab [19]. The above two reports concerned the association between statins and the efficacy of cancer immunotherapy. However, the authors conducted the analyses without PD-L1 data, which is the main prognostic and predictive marker for cancer immunotherapy in the clinical setting, and with a small sample size. Therefore, the clinical impact of statin therapy on the outcome of cancer immunotherapy in patients with NSCLC is poorly understood.

We investigated the clinical impact of statin therapy on the survival of patients with NSCLC treated with anti-PD-1 monotherapy. In this multicenter and retrospective study, we performed a propensity score-matched analysis to minimize the bias arising from the patients’ backgrounds.

## Methods

### Patients enrolled in this study

We conducted this retrospective study in accordance with the amended Declaration of Helsinki, and it was approved by our institutional review boards (Kyushu University, IRB No. 2020-76; National Hospital Organization Kyushu Cancer Center, IRB No. 2019-45; and Kitakyushu Municipal Medical Center, IRB No. 202008008). The requirement of informed consent from the patients enrolled in this study was waived because of the retrospective design, and patient information was protected.

**Fig. 1:**
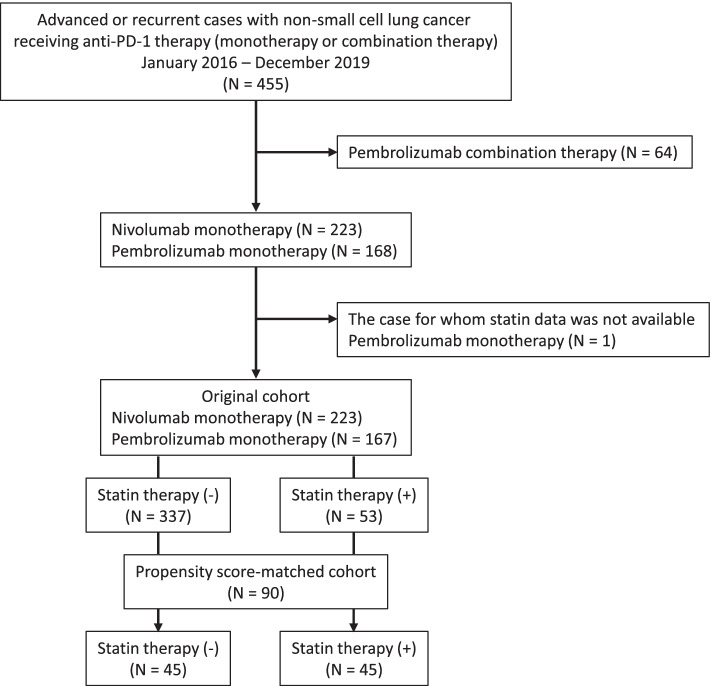
CONSORT diagram of this study. PD-1, programmed cell death-1

The above 3 institutions participated to this retrospective study, and total 455 consecutive patients with advanced or recurrent NSCLC treated with anti-PD-1 therapy (monotherapy or combination therapy) in clinical practice between 2016 − 2019 were identified in our database. Of these, we excluded 64 patients treated with pembrolizumab combination therapy and 1 patient for whom statin data were not available. We therefore ultimately enrolled 390 patients, as shown in Fig. 1.

Nivolumab and pembrolizumab were administered intravenously at a dose of 3 mg/kg every 2 weeks and at a fixed dose of 200 mg every 3 weeks, respectively. Moreover, the patients did not receive other cancer-related treatments except cancer immunotherapy. The variables investigated in this study were the age (continuous variable), biological sex (female vs. male), Eastern Cooperative Oncology Group (ECOG) performance status (PS) (0 vs. 1 − 3), smoking (never-smoker vs. smoker), checkpoint inhibitor (nivolumab vs. pembrolizumab), treatment line (first vs. second or later), histology (non-sq vs. sq), stage (advanced vs. recurrent), body mass index (BMI) (< 22 vs. ≥ 22), driver gene mutation (others vs. wild-type), PD-L1 expression (others vs. tumor proportion score [TPS] ≥ 50%), and presence of statin therapy (no vs. yes). The BMI was calculated from the height and weight measured at the time of treatment initiation. Statins included atorvastatin, pitavastatin, pravastatin, rosuvastatin, and simvastatin; any use at the time of treatment initiation as a daily use medicine was examined in this study, regardless of the dose and duration. The PD-L1 status and epidermal growth factor receptor (*EGFR*)/anaplastic lymphoma kinase (*ALK*) status were evaluated in accordance with the assay manufacturers’ recommended methods [20–22]. We obtained all clinical information, including the PD-L1, *EGFR*, and *ALK* status, and follow-up data from patients’ medical records.

### Statistical analyses

We conducted all statistical analyses in this study using the JMP® 14.0 or SAS® 9.4 software programs (SAS Institute, Cary, NC, USA) and considered *P* <  0.05 statistically significant. We analyzed the relationships between statin therapy and patient characteristics using independent *t*-tests for continuous variables and Pearson’s chi-squared test for categorical variables. We defined the PFS and overall survival (OS) as previously reported [23]. We constructed the survival curves using the Kaplan–Meier method with the log-rank test. A Cox proportional hazards regression analysis was used to estimate the hazard ratios (HRs) for risk factors, and we used the backward elimination method in the multivariate analysis as previously reported [23]. We also conducted the propensity score-matched analysis using the JMP 14.0 or SAS 9.4 software programs (SAS Institute, Cary, NC, USA). Propensity score-matching was performed with the use of 1:1 matching without replacement (greedy-matching algorithm), with a caliper width equal to 0.1 of the standard deviation of the logit of the propensity score. Standardized mean differences were estimated for all baseline covariates before and after matching to assess the prematch imbalance and postmatch balance, and a standardized mean difference of < 0.25 indicated a relatively small imbalance in this study [24–26]. Survival analyses using the Kaplan–Meier method with the log-rank test and Cox proportional hazards regression analysis were conducted to compare the matched pairs.

## Results

### Patient characteristics in the original cohort

Table 1 shows the clinical characteristics of the 390 patients enrolled in this study. The median age was 67 (range, 31 − 88) years old, and 309 (79.2%) patients were men. Among the 390 patients, 53 (13.6%) received statin therapy, including atorvastatin in 12, pitavastatin in 10, pravastatin in 9, rosuvastatin in 19, and simvastatin in 3. Data on the *EGFR* or *ALK* status were available for 326 patients (83.6%), and PD-L1 data were available for 261 patients (66.9%).

**Table 1:** Clinicopathological characteristics of all patients (*N* = 390)

Characteristic	Value or ***N*** (%)
Age (years)	
Median	67
Range	31 − 88
Sex	
Female	81 (20.8%)
Male	309 (79.2%)
ECOG PS	
0	144 (36.9%)
1	213 (54.6%)
2	28 (7.2%)
3	5 (1.3%)
Line of treatment	
First	95 (24.4%)
Second	121 (31.0%)
Third or higher	174 (44.6%)
Smoking history	
Never-smoker	68 (17.4%)
Ex-smoker	196 (50.3%)
Current smoker	126 (32.3%)
Clinical stage	
Advanced	305 (78.2%)
Recurrent	85 (21.8%)
Mutation status (*EGFR* or *ALK*)	
Wild-type	280 (71.8%)
Mutation^a^	46 (11.8%)
Unknown	64 (16.4%)
Histology	
Adenocarcinoma	249 (63.8%)
Squamous cell carcinoma	106 (27.2%)
Others or unknown^b^	35 (9.0%)
Immune checkpoint inhibitor	
Nivolumab	223 (57.2%)
Pembrolizumab	167 (42.8%)
PD-L1 tumor proportion score	
< 1%	51 (13.1%)
≥ 1 and < 50%	82 (21.0%)
≥ 50%	128 (32.8%)
Unknown	129 (33.1%)
Body mass index (kg/m^2^)	
< 22	213 (54.6%)
≥ 22	177 (45.4%)
Statin therapy	
No	337 (86.4%)
Yes	53 (13.6%)

*ALK* anaplastic lymphoma kinase, *ECOG* Eastern Cooperative Oncology Group, *EGFR* epidermal growth factor receptor, *PD-L1* programmed cell death-ligand 1, *PS* performance status

^a^Among 46 patients, 42 patients were *EGFR*-positive and four patients were *ALK*-positive

^b^Among 35 patients, 11 patients had sarcomatoid carcinoma, 23 patients had not-otherwise specified, and one patient had adenosquamous carcinoma

Table 2 summarizes the baseline characteristics of the patients according to statin therapy. As shown in Table 2, the use or non-use of statins was associated with the age, sex, smoking history, BMI, and mutation status in the original cohort (*P* <  0.0001, *P* = 0.0036, *P* = 0.0085, *P* = 0.0032, and *P* = 0.0508, respectively; Table 2).

**Table 2:** Characteristics of patients according to statin therapy before and after propensity score matching

Characteristic	Statin therapy	Before matching, ***N*** (%)				After matching, ***N*** (%)			
		No (***N*** = 337)	Yes (***N*** = 53)	***P***-value	SMD	No (***N*** = 45)	Yes (***N*** = 45)	***P***-value	SMD
Age (years)	Mean (SD)	65.1 (10.0)	71.6 (7.8)	< 0.0001	0.7272	70.8 (8.3)	70.9 (8.0)	0.9589	0.0109
Sex	Female	62 (18.4%)	19 (35.9%)	0.0036	−0.4003	16 (35.6%)	13 (28.9%)	0.4986	0.1430
	Male	275 (81.6%)	34 (64.1%)			29 (64.4%)	32 (71.1%)		
ECOG PS	0	125 (37.1%)	19 (35.9%)	0.8616	−0.0258	13 (28.9%)	17 (37.8%)	0.3711	0.1894
	1–3	212 (62.9%)	34 (64.1%)			32 (71.1%)	28 (62.2%)		
Smoking history	Never-smoker	52 (15.4%)	16 (30.2%)	0.0085	0.3573	14 (31.1%)	12 (26.7%)	0.6418	−0.0982
	Smoker	285 (84.6%)	37 (69.8%)			31 (68.9%)	33 (73.3%)		
Immune checkpoint inhibitor	Nivolumab	196 (58.2%)	27 (50.9%)	0.3236	−0.1453	25 (55.6%)	21 (46.7%)	0.3990	−0.1785
	Pembrolizumab	141 (41.8%)	26 (49.1%)			20 (44.4%)	24 (53.3%)		
Line of treatment	First	78 (23.2%)	17 (32.1%)	0.1592	0.2008	14 (31.1%)	15 (33.3%)	0.8215	0.0476
	Second or higher	259 (76.8%)	36 (67.9%)			31 (68.9%)	30 (66.7%)		
Histology	Non-Sq	243 (72.1%)	41 (77.4%)	0.4244	0.1211	34 (75.6%)	34 (75.6%)	1.0000	0.0000
	Sq	94 (27.9%)	12 (22.6%)			11 (24.4%)	11 (24.4%)		
Clinical stage	Advanced	262 (77.7%)	43 (81.1%)	0.5787	0.0839	34 (75.6%)	36 (80.0%)	0.6121	0.1071
	Recurrent	75 (22.3%)	10 (18.9%)			11 (24.4%)	9 (20.0%)		
Body mass index (kg/m^2^)	< 22	194 (57.6%)	19 (35.9%)	0.0032	−0.4460	23 (51.1%)	17 (37.8%)	0.2031	−0.2708
	≥ 22	143 (42.4%)	34 (64.1%)			22 (48.9%)	28 (62.2%)		
Mutation status (*EGFR* or *ALK*)	Others^a^	101 (30.0%)	9 (17.0%)	0.0508	0.3101	4 (8.9%)	8 (17.8%)	0.2148	−0.2638
	Wild-type	236 (70.0%)	44 (83.0%)			41 (91.1%)	37 (82.2%)		
PD-L1 tumor proportion score	Others^b^	228 (67.7%)	34 (64.1%)	0.6135	0.0740	30 (66.7%)	28 (62.2%)	0.6596	0.0930
	≥ 50%	109 (32.3%)	19 (35.9%)			15 (33.3%)	17 (37.8%)		

*ALK* anaplastic lymphoma kinase, *ECOG* Eastern Cooperative Oncology Group, *EGFR* epidermal growth factor receptor, *PD-L1* programmed cell death-ligand 1, *PS* performance status, *SD* standard deviation, *SMD* standardized mean difference, *Sq* squamous cell carcinoma

^a^Mutation plus unknown

^b^ < 50% or unknown

### Characteristics of patients according to statin therapy after propensity score matching

Propensity score matching was conducted as described in the statistical methods. The propensity scores, calculated by a multivariate logistic analysis, included the following factors: age, sex, smoking history, BMI, and mutation status. The 45 matched patients from the statin and non-statin groups were included in a propensity score-matched analysis (Fig. 1). As described in the statistical methods, standardized mean differences were estimated for all baseline covariates before and after matching to assess the prematch imbalance and postmatch balance, and a standardized mean difference of < 0.25 indicated a relatively small imbalance in this study. The standardized mean differences of the whole model before and after propensity score matching were 0.9621 and 0.1427, respectively. After propensity score matching, the baseline patient characteristics between the two groups were well-balanced, as shown in Table 2.

### Results of the survival analysis in the original cohort

First, we investigated the effects of statin therapy on the survival in the original cohort. The median follow-up time was 416 days (range, 3–1701). No patients died from any disease other than lung cancer in this study. Kaplan–Meier curves revealed no significant differences in the PFS or OS between patients who did and did not receive statin therapy (*P* = 0.4777 and *P* = 0.5264, respectively; Supplementary Fig. 1a and b). Multivariate analyses revealed that the ECOG PS (PS 1 − 3 vs. PS 0: HR = 1.36, *P* = 0.0084), smoking history (never-smoker vs. smoker: HR = 1.37, *P* = 0.0298), and PD-L1 expression status (others vs. ≥ TPS 50%: HR = 1.64, *P* < 0.0001) were independent prognostic factors for the PFS (Supplementary Table 1), whereas the ECOG PS (PS 1 − 3 vs. PS 0: HR = 1.66, *P* = 0.0001) and PD-L1 expression status (others vs. ≥ TPS 50%: HR = 1.52, *P* = 0.0026) were independent prognostic factors for the OS (Supplementary Table 1).

### Results of the survival analysis in the propensity score-matched cohort

**Fig. 2:**
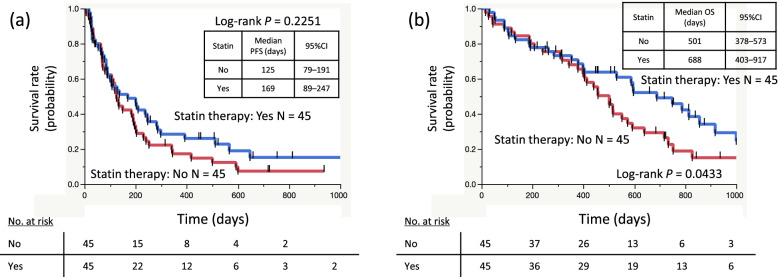
Kaplan–Meier curves of (**a**) progression-free survival and (**b**) overall survival according to statin therapy in the propensity score-matched cohort. CI, confidence interval; OS, overall survival; PFS, progression-free survival

Next, we investigated the effects of statin therapy on the survival in the propensity score-matched cohort. The median follow-up time was 457 days (range, 15–1358). Kaplan–Meier curves showed that patients who received statin therapy had a significantly longer OS (*P* = 0.0433), but not PFS (*P* = 0.2251), than those who did not receive statin therapy (Fig. 2a and b). Cox analyses showed that ICI use was an independent prognostic factor for the PFS (nivolumab vs. pembrolizumab: HR = 2.07, *P* = 0.0021; Table 3), whereas the histology (Sq vs. non-Sq: HR = 1.80, *P* = 0.0337) and PD-L1 expression status (others vs. ≥ TPS 50%: HR = 2.29, *P* = 0.0052) were independent prognostic factors for the OS (Table 3). A Cox regression analysis in the propensity score-matched cohort showed that the use of statin therapy was not an independent favorable prognostic factor, although it tended to be correlated with a favorable outcome (use vs. non-use: HR = 0.61, *P* = 0.0585; Table 3).

**Table 3:** Univariate and multivariate analyses of PFS and OS in the propensity score-matched cohort

Characteristics		PFS				OS			
		Univariate analysis		Multivariate analysis		Univariate analysis		Multivariate analysis	
		HR (95%CI)	***P***-value	HR (95%CI)	***P***-value	HR (95%CI)	***P***-value	HR (95%CI)	***P***-value
Age (years)	Continuous variable	0.98 (0.95 − 1.01)	0.1148			0.98 (0.94 − 1.01)	0.1984		
Sex	Female/male	0.94 (0.58 − 1.54)	0.8097			0.73 (0.42 − 1.26)	0.2574		
ECOG PS	1 − 3/0	1.44 (0.89 − 2.33)	0.1391			1.46 (0.85 − 2.51)	0.1747		
Smoking history	Never-smoker/smoker	1.00 (0.61 − 1.63)	0.9876			0.74 (0.43 − 1.30)	0.2985		
Immune checkpoint inhibitor	Nivolumab/pembrolizumab	2.10 (1.33 − 3.34)	0.0016	2.07 (1.30 − 3.28)	0.0021	2.31 (1.38 − 3.87)	0.0015		
Line of treatment	Second or higher/first	1.68 (1.01 − 2.78)	0.0440			1.74 (0.97 − 3.12)	0.0613		
Histology	Sq/non-Sq	1.62 (0.98 − 2.67)	0.0590	1.55 (0.94 − 2.57)	0.0849	1.57 (0.92 − 2.67)	0.0954	1.80 (1.05 − 3.09)	0.0337
Clinical stage	Advanced/recurrent	1.25 (0.72 − 2.16)	0.4332			0.90 (0.48 − 1.68)	0.7433		
Body mass index (kg/m^2^)	< 22/≥22	0.90 (0.58 − 1.42)	0.6637			0.84 (0.51 − 1.38)	0.4834		
Mutation status (*EGFR* or *ALK*)	Others^a^ /wild-type	1.02 (0.54 − 1.93)	0.9529			0.85 (0.41 − 1.73)	0.6498		
PD-L1 tumor proportion score	Others^b^/≥50%	1.90 (1.17 − 3.10)	0.0100			2.36 (1.33 − 4.18)	0.0033	2.29 (1.28 − 4.08)	0.0052
Statin therapy	Yes/no	0.76 (0.48 − 1.19)	0.2277			0.60 (0.36 − 0.99)	0.0456	0.61 (0.36 − 1.02)	0.0585

*ALK* anaplastic lymphoma kinase, *CI* confidence interval, *ECOG* Eastern Cooperative Oncology Group, *EGFR* epidermal growth factor receptor, *HR* hazard ratio, *OS* overall survival, *PD-L1* programmed cell death-ligand 1, *PFS* progression-free survival, *PS* performance status, *Sq* squamous cell carcinoma

^a^Mutation plus unknown

^b^ < 50% or unknown

## Discussion

In this multicenter and retrospective study, no significant differences in the PFS and OS were observed between NSCLC patients with and without statin treatment in the original cohort. However, the patient characteristics of NSCLC patients with statin therapy were associated with the age, sex, smoking history, BMI, and mutation status (Table 2), findings that were similar to those previously reported [27]. After these biases were adjusted for by propensity score matching, NSCLC patients with statin therapy had a significantly longer OS than those without statin therapy. Thus, our findings suggested that the use of statins might contribute to a favorable prognosis in NSCLC patients treated with anti-PD-1 monotherapy.

With regard to independent prognostic factors of the PFS in the original cohort, the ECOG PS, smoking history, and PD-L1 were selected, and ECOG PS and PD-L1 were also independent prognostic factors of the OS (Supplementary Table 1). The ECOG PS, smoking history, and PD-L1 were all previously reported to be significant predictors of the efficacy of ICIs in NSCLC patients [28–31]. Our results were in line with those of previous reports, suggesting that our findings might be applicable to a general NSCLC population receiving ICIs.

Statins inhibit HMG-CoA reductase and block the rate-limiting enzyme of the mevalonate pathway [32]. The mevalonate pathway is an essential metabolic pathway for cholesterol biosynthesis in the liver [33]. Statins are the most common cholesterol-lowering drugs and contribute to a reduced risk of illnesses related to atherosclerosis. Interestingly, several previous studies have suggested that statin use is associated with improved clinical outcomes in patients with cancer through the mevalonate pathway [34–39]. Nielsen et al. reported that statin use before a cancer diagnosis contributed to a statistically significant reduction of 15% in all-cancer mortality [14]. Regarding thoracic malignancies, a few previous studies have indicated the clinical impact of statins on the efficacy of ICIs [18, 19]. Cantini et al. reported that baseline statin use was significantly related to an improved response rate, PFS, and OS in malignant pleural mesothelioma and NSCLC patients treated with PD-1 inhibitors [18]. Omori et al. also indicated that statins significantly improved the response rates and prolonged the time-to-treatment failure in NSCLC patients treated with nivolumab [19]. Our results were at least partly in line with these reports. The results of the current study showed that the patients who received statin therapy had a significantly longer OS, but not PFS, than those who did not receive statin therapy. However, this study included 390 patients, which was the largest cohort among the studies that investigated the clinical impact of statin therapy on the survival of patients with NSCLC receiving cancer immunotherapy. Moreover, a propensity score-matched analysis was conducted to minimize the bias arising from the patients’ backgrounds in this study, which was not conducted in the previous two studies. At the same time, we did not examine the relationship between statin therapy and tumor response in patients with NSCLC receiving cancer immunotherapy because only 45 matched patients from the statin and non-statin groups were included in the propensity score-matched analysis. We should validate the findings in further prospective studies with a larger sample size.

Previous studies have indicated the mechanisms underlying the clinical effects of statins on tumor biology and immunomodulatory properties [40]. Statins have the ability to trigger tumor-specific apoptosis by inhibiting geranylgeranylation of Rho proteins [40]. Inhibiting the mevalonate pathway by statins also enhances antigen presentation, prolongs antigen retention, and activates T cells by blocking the geranylgeranylation of small GPTase [41]. Lipophilic statins are reported to enhance antigen-specific antitumor immunity (Th1 and cytolytic T cell responses) [41]. In addition, pre-clinical studies suggested that blocking the mevalonate pathway has a direct antitumor effect by interacting with oncogenic molecules, including p53, Myc, and phosphatidylinositol 3-kinase [42]. Although the mechanisms underlying our findings were not analyzed in this study, this previously reported evidence may explain why the use of statins resulted in a prolonged OS in NSCLC patients treated with anti-PD-1 monotherapy. Moreover, there might be another possible mechanism underlying the effect of statin use on clinical outcome in patients with NSCLC receiving cancer immunotherapy. Recently, several studies have revealed the influence of the gastrointestinal microbiota on the response to cancer immunotherapy [43–45]. Drugs associated with gastrointestinal dysbiosis and bacterial richness, such as antibiotics, proton pump inhibitors, and probiotics, might affect the efficacy of ICIs in NSCLC patients [6, 8]. A recent study showed that statin therapy was also associated with a lower prevalence of gut microbiota dysbiosis [46]. From these findings, statin therapy might be associated with the efficacy of ICIs in NSCLC patients. Further additional translational studies investigating the biological relationship between statins and the efficacy of PD-1 inhibitors are warranted.

Kaplan–Meier curves showed that patients who received statin therapy had significantly longer OS than those who did not receive statin therapy. The Kaplan–Meier curves part after approximately 400 days, which is much longer than median PFS. Several preclinical studies showed that statins might have a synergic effect in combination with cytotoxic chemotherapy, not immunotherapy, in solid tumors [47–49]. However, some meta-analyses of randomized controlled trials of statin therapy added to systemic anticancer therapy in solid tumors indicated that this combination had no clinical benefits [50, 51]. Therefore, whether statin therapy had a positive effect on the efficacy of subsequent treatment or not was unknown.

Several limitations associated with the present study warrant mention. First, we did not analyze the type, intensity, or lipophilicity (lipophilic or hydrophilic) of the statins because of the small number of patients receiving statins (*N* = 53). According to a previous report, although the use of high-intensity statins was significantly associated with better clinical outcomes, there were no marked differences in the efficacy of ICIs between patients taking low−/moderate-intensity statins and those who were not taking such medication [18]. A further detailed analysis of the clinical impact of statin types, intensity, and lipophilicity on the efficacy of ICIs is necessary. Second, there was a heterogeneity of the included patients such as recurrent or advanced cases and adenocarcinoma, squamous cell carcinoma or other types of histology in this study. Therefore, we should interpret the study results with caution in this point. Third, this was a translational study associated with some bias due to the retrospective nature of this study. Some patients in this study may have suffered from other chronic diseases such as diabetes and heart disease, regularly receiving antidiabetic drugs or heart disease drugs such as metformin and beta blockers, which have a potential impact on the efficacy of cancer immunotherapy in patients with NSCLC [5, 9]. However, we do not have these data and cannot conduct subgroup analyses according to these chronic diseases. Further studies including information about the abovementioned factors may also be warranted. Fourth, we categorized PS into 0 or 1–3, which seemed incorrect. If we categorized PS into 0 or 1–3, the proportion of PS 3 was unbalanced between the two groups. We think that it is better to categorize PS as 0/1 or 2/3. However, we could not conduct such statistical analyses including a propensity score-matched analysis if we categorized PS as 0/1 or 2/3 because of small number of the patient who received statin therapy and had a PS of 2/3 (*N* = 1). In our previous studies, we categorized PS into 0 or 1–3 [52–54]. Therefore, we also categorized PS into 0 or 1–3 in this study.

## Conclusions

In conclusion, statin therapy might be a combination tool for cancer immunotherapy in patients with NSCLC. These findings should be validated in further prospective studies with larger sample sizes.

## Supplementary Information

**Additional file 1: Supplementary Figure 1.** Kaplan–Meier curves of (a) progression-free survival and (b) overall survival according to statin therapy in the original cohort. CI, confidence interval; OS, overall survival; PFS, progression-free survival.

**Additional file 2: Supplementary Table 1.** Univariate and multivariate analyses of PFS and OS in the original cohort.

## Data Availability

All data and materials are available in this study, and the data that support the findings of this study are available from the corresponding author upon reasonable request.

## Abbreviations

ALK: Anaplastic lymphoma kinase
BMI: Body mass index
CI: Confidence interval
ECOG: Eastern Cooperative Oncology Group
EGFR: Epidermal growth factor receptor
HMG-CoA: 3-hydroxy-3-methylglutaryl coenzyme A
HR: Hazard ratio
ICIs: Immune checkpoint inhibitors
NSCLC: Non-small cell lung cancer
OS: Overall survival
PD-1: Programmed cell death-1
PD-L1: Programmed cell death-ligand 1
PFS: Progression-free survival
PS: Performance status
TPS: Tumor proportion score
