# Supplementary material for: A propensity score-matched analysis of the impact of statin therapy on the outcomes of patients with non-small-cell lung cancer receiving anti-PD-1 monotherapy: a multicenter retrospective study
Source: BMC Cancer. 2022 May 6;22:503. doi: 10.1186/s12885-022-09385-8 (PMC9074359; doi:10.1186/s12885-022-09385-8)
Supplement: Supplementary file 1 — Additional file 1: Supplementary Figure 1. Kaplan–Meier curves of (a) progression-free survival and (b) overall survival according to statin therapy in the original cohort. CI, confidence interval; OS, overall survival; PFS, progression-free survival. [file 12885_2022_9385_MOESM1_ESM.pptx]

## Slide 1
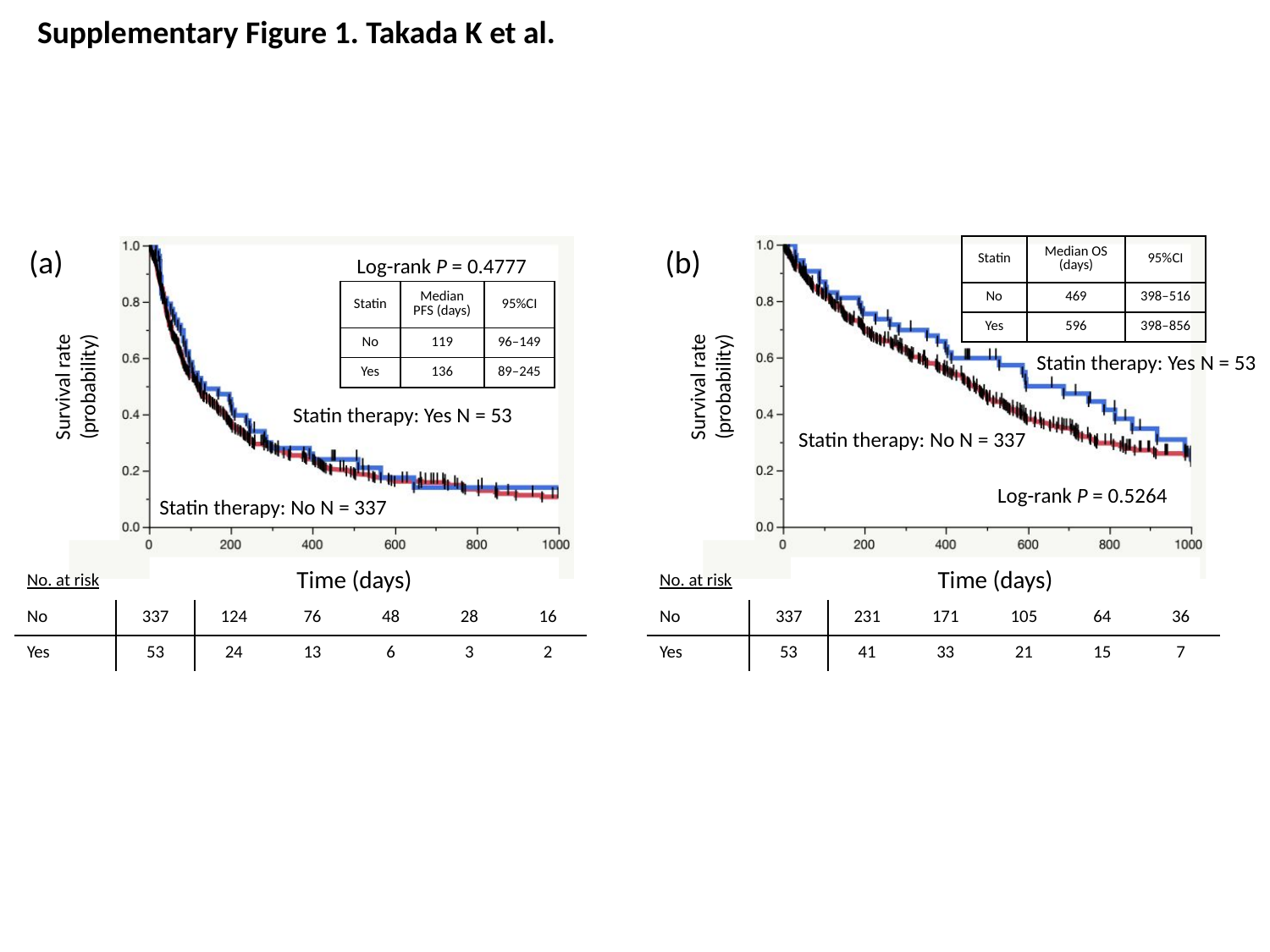

Supplementary Figure 1. Takada K et al.
Survival rate
(probability)
Survival rate
(probability)
| Statin | Median OS (days) | 95%CI |
| --- | --- | --- |
| No | 469 | 398–516 |
| Yes | 596 | 398–856 |
(a)
(b)
Log-rank P = 0.4777
| Statin | Median PFS (days) | 95%CI |
| --- | --- | --- |
| No | 119 | 96–149 |
| Yes | 136 | 89–245 |
Statin therapy: Yes N = 53
Statin therapy: Yes N = 53
Statin therapy: No N = 337
Log-rank P = 0.5264
Statin therapy: No N = 337
Time (days)
Time (days)
| No. at risk | | | | | | |
| --- | --- | --- | --- | --- | --- | --- |
| No | 337 | 124 | 76 | 48 | 28 | 16 |
| Yes | 53 | 24 | 13 | 6 | 3 | 2 |
| No. at risk | | | | | | |
| --- | --- | --- | --- | --- | --- | --- |
| No | 337 | 231 | 171 | 105 | 64 | 36 |
| Yes | 53 | 41 | 33 | 21 | 15 | 7 |
