# Supplementary material for: A propensity score-matched analysis of the impact of statin therapy on the outcomes of patients with non-small-cell lung cancer receiving anti-PD-1 monotherapy: a multicenter retrospective study
Source: BMC Cancer. 2022 May 6;22:503. doi: 10.1186/s12885-022-09385-8 (PMC9074359; doi:10.1186/s12885-022-09385-8)
Supplement: Supplementary file 2 — Additional file 2: Supplementary Table 1. Univariate and multivariate analyses of PFS and OS in the original cohort. [file 12885_2022_9385_MOESM2_ESM.doc]

**Supplementary Table 1.** Univariate and multivariate analyses of PFS and OS in the original cohort

| **Characteristics** |  | **PFS** | | | | |  | **OS** | | | | |
| --- | --- | --- | --- | --- | --- | --- | --- | --- | --- | --- | --- | --- |
|  |  | **Univariate analysis** | |  | **Multivariate analysis** | |  | **Univariate analysis** | |  | **Multivariate analysis** | |
|  |  | **HR (95%CI)** | ***P*-value** |  | **HR (95%CI)** | ***P*-value** |  | **HR (95%CI)** | ***P*-value** |  | **HR (95%CI)** | ***P*-value** |
| Age (years) | Continuous variable | 1.00 (0.99−1.01) | 0.9775 |  |  |  |  | 1.00 (0.99−1.02) | 0.5721 |  |  |  |
|  |  |  |  |  |  |  |  |  |  |  |  |  |
|  |  |  |  |  |  |  |  |  |  |  |  |  |
| Sex | Female/male | 1.27 (0.98−1.66) | 0.0729 |  |  |  |  | 1.15 (0.86−1.54) | 0.3330 |  |  |  |
|  |  |  |  |  |  |  |  |  |  |  |  |  |
|  |  |  |  |  |  |  |  |  |  |  |  |  |
| ECOG PS | 1−3/0 | 1.48 (1.18−1.85) | 0.0007 |  | 1.36 (1.08−1.71) | 0.0084 |  | 1.72 (1.33−2.23) | < 0.0001 |  | 1.66 (1.28−2.15) | 0.0001 |
|  |  |  |  |  |  |  |  |  |  |  |  |  |
|  |  |  |  |  |  |  |  |  |  |  |  |  |
| Smoking history | Never-smoker/  smoker | 1.41 (1.06−1.86) | 0.0168 |  | 1.37 (1.03−1.81) | 0.0298 |  | 1.21 (0.89−1.65) | 0.2217 |  |  |  |
|  |  |  |  |  |  |  |  |  |  |  |  |  |
|  |  |  |  |  |  |  |  |  |  |  |  |  |
| Immune checkpoint inhibitor | Nivolumab/  pembrolizumab | 1.57 (1.26−1.95) | < 0.0001 |  |  |  |  | 1.45 (1.13−1.86) | 0.0038 |  |  |  |
|  |  |  |  |  |  |  |  |  |  |  |  |  |
|  |  |  |  |  |  |  |  |  |  |  |  |  |
| Line of treatment | Second or higher/first | 1.67 (1.28−2.18) | 0.0002 |  |  |  |  | 1.63 (1.20−2.22) | 0.0018 |  |  |  |
|  |  |  |  |  |  |  |  |  |  |  |  |  |
|  |  |  |  |  |  |  |  |  |  |  |  |  |
| Histology | Sq/non-Sq | 1.10 (0.87−1.40) | 0.4231 |  |  |  |  | 1.19 (0.92−1.55) | 0.1893 |  |  |  |
|  |  |  |  |  |  |  |  |  |  |  |  |  |
|  |  |  |  |  |  |  |  |  |  |  |  |  |
| Clinical stage | Advanced/  recurrent | 1.07 (0.82−1.39) | 0.6081 |  |  |  |  | 1.17 (0.86−1.59) | 0.3134 |  |  |  |
|  |  |  |  |  |  |  |  |  |  |  |  |  |
|  |  |  |  |  |  |  |  |  |  |  |  |  |
| Body mass index (kg/m^2^) | <22/≥22 | 1.01 (0.82−1.25) | 0.9114 |  |  |  |  | 1.11 (0.87−1.41) | 0.4045 |  |  |  |
|  |  |  |  |  |  |  |  |  |  |  |  |  |
|  |  |  |  |  |  |  |  |  |  |  |  |  |
| Mutation status (*EGFR* or *ALK*) | Others^a^ /  wild-type | 1.36 (1.07−1.72) | 0.0115 |  |  |  |  | 1.38 (1.06−1.79) | 0.0166 |  |  |  |
|  |  |  |  |  |  |  |  |  |  |  |  |  |
|  |  |  |  |  |  |  |  |  |  |  |  |  |
| PD-L1 tumor proportion score | Others^b^/≥50% | 1.70 (1.34−2.16) | < 0.0001 |  | 1.64 (1.29−2.08) | < 0.0001 |  | 1.60 (1.22−2.10) | 0.0007 |  | 1.52 (1.16−2.00) | 0.0026 |
|  |  |  |  |  |  |  |  |  |  |  |  |  |
|  |  |  |  |  |  |  |  |  |  |  |  |  |
|  |  |  |  |  |  |  |  |  |  |  |  |  |
| Statin therapy | Yes/no | 0.89 (0.65−1.23) | 0.4791 |  |  |  |  | 0.89 (0.63−1.27) | 0.5270 |  |  |  |
|  |  |  |  |  |  |  |  |  |  |  |  |  |

^a^Mutation plus unknown

^b^< 50% or unknown

*ALK*, anaplastic lymphoma kinase; CI, confidence interval; ECOG, Eastern Cooperative Oncology Group; *EGFR*, epidermal growth factor receptor; HR, hazard ratio; OS, overall survival; PD-L1, programmed cell death-ligand 1; PFS, progression-free survival; PS, performance status; Sq, squamous cell carcinoma.
